# Dauricine Attenuates Vascular Endothelial Inflammation Through Inhibiting NF-κB Pathway

**DOI:** 10.3389/fphar.2021.758962

**Published:** 2021-12-01

**Authors:** Ji Hu, Ru Chen, Jie An, Yilong Wang, Minglu Liang, Kai Huang

**Affiliations:** ^1^ Department of Cardiology, Union Hospital, Tongji Medical College, Huazhong University of Science and Technology, Wuhan, China; ^2^ Clinic Center of Human Gene Research, Union Hospital, Tongji Medical College, Huazhong University of Science and Technology, Wuhan, China; ^3^ Department of Cardiology, Handan First Hospital, Handan, China; ^4^ Department of Cardiology, The First Affiliated Hospital of Sun Yat-Sen University, Guangzhou, China; ^5^ Hubei Key Laboratory of Metabolic Abnormalities and Vascular Aging, Huazhong University of Science and Technology, Wuhan, China; ^6^ Hubei Clinical Research Center of Metabolic and Cardiovascular Disease, Huazhong University of Science and Technology, Wuhan, China

**Keywords:** dauricine, endothelial dysfunction, NF-κB pathway, acute lung injury, inflammation

## Abstract

Endothelial cells are the fundamental components of blood vessels that regulate several physiological processes including immune responses, angiogenesis, and vascular tone. Endothelial dysfunction contributes to the development of various diseases such as acute lung injury, and endothelial inflammation is a vital part of endothelial dysfunction. Dauricine is an extract isolated from *Menispermum dauricum* DC, a traditional Chinese medical plant that can be used for pharyngitis. In this work, we found that IL-1β-induced overexpression of intercellular adhesion molecule-1 (ICAM-1), vascular cell adhesion molecule-1 (VCAM-1), and E-selectin was inhibited by dauricine in primary human umbilical vein endothelial cells (HUVECs). Correspondingly, adhesion of human acute monocytic leukemia cell line (THP-1) to HUVECs was decreased by dauricine. Further studies showed that dauricine inhibited the activation of nuclear factor-κB (NF-κB) pathway in HUVECs stimulated with IL-1β. *In vivo*, dauricine protected mice from lipopolysaccharide (LPS)-induced acute lung injury. In lung tissues, the activation of NF-κB pathway and the expression of its downstream genes (ICAM-1, VCAM-1, and E-selectin) were decreased by dauricine, consistent with what was found *in vitro*. In summary, we concluded that dauricine could alleviate endothelial inflammation by suppressing NF-κB pathway, which might serve as an effective candidate for diseases related with endothelial inflammation.

## Introduction

The vascular endothelium forms the inner surface of the cardiovascular system. It is not only a natural blood–organ barrier but also an endocrine tissue that plays pivotal roles in immune responses, angiogenesis, hemostasis, and the regulation of vascular tone (Boulanger, 2016; Sturtzel, 2017). Endothelial dysfunction has been noticed in various pathological states, including atherosclerosis, hypertension, kidney disease, and sepsis (Gimbrone and García-Cardeña, 2016; Konukoglu and Uzun, 2017; Jourde-Chiche et al., 2019; Joffre et al., 2020). Aggravated endothelial inflammation, which is characterized by overexpressed cytokines and adhesion molecules such as intercellular adhesion molecule-1 (ICAM-1), vascular cell adhesion molecule-1 (VCAM-1), and selectins (Konukoglu and Uzun, 2017), is an important pathological process in endothelial dysfunction. Several molecules and pathways that regulate endothelial inflammation have been elucidated, among which the classic nuclear factor-κB (NF-κB) proinflammatory pathway attracted wide attention (Brasier, 2010; Rao et al., 2019).

Dauricine is an alkaloid extracted from the roots of *Menispermum dauricum* DC, and this compound is a traditional Chinese medicine that has been used to treat rheumatism (Du et al., 1986). In early studies, dauricine was found to exert an inhibitory effect in several inflammatory mouse models (Du et al., 1986). Later, multiple functions of dauricine were reported. Dauricine inhibited viability and induced cell apoptosis by inhibiting the PI3K/Akt pathway in renal carcinoma cells (Zhang et al., 2018). Lipopolysaccharide (LPS)-induced bone loss was inhibited by dauricine as mediated via the ROS/PP2A/NF-κB axis (Park et al., 2020). In a cerebral ischemia/reperfusion rat model, dauricine attenuated the inflammatory process by downregulating the expression of ICAM-1, TNF-α, and IL-1β (Yang et al., 2007). Studies performed both *in vivo* and *in vitro* have proven that dauricine protected against *Streptococcus pneumoniae* coinfected with influenza virus H5N1 through NF-κB pathway (Li et al., 2018). In our previous work, we found that dauricine negatively regulated LPS-induced acute lung injury and immune response of macrophages (Qiao et al., 2019). However, the effect of dauricine on endothelial inflammation remains unclear.

Since endothelial inflammation is involved in multiple inflammatory diseases, targeting the inflammatory process in endothelial cells may provide a potential therapeutic strategy. Dauricine, an anti-inflammatory drug that has been proven effective in treating the aforementioned inflammatory diseases, might be an effective inhibitor of endothelial inflammation. In this work, we intended to discover the function and potential mechanism of dauricine in endothelial inflammation.

## Materials and Methods

### Cell Culture

Human umbilical vein endothelial cells (HUVECs) were cultured in specific endothelial medium (ScienCell, Carlsbad, CA, United States) containing 1% endothelial cell growth supplement, 5% fetal bovine serum, and 1% penicillin-streptomycin solution in a cell incubator (37°C with 5% CO_2_). *In vitro* experiments were performed on HUVECs between passages 2 and 6. The human leukemia monocytic cell line (THP-1) and human embryonic kidney-293T (293T) cell line were obtained from American Type Culture Collection (ATCC, Manassas, VA, United States).

### Reagents

LPS was purchased from Sigma (St. Louis, MO, United States). Dauricine was obtained from Shanghai Yuanye Bio-Technology Co., Ltd (Shanghai, China) and diluted in dimethyl sulfoxide (DMSO). Four concentrations (5, 10, 20, and 40 μM) were finally reached in further experiment (Yang et al., 2010). The solvent DMSO was used as vehicle control, and its final concentration in the cell culture medium was controlled to be less than 0.1%. Ultrafiltered water was used to dissolve the interleukin-1β (final concentration, 10 ng/μl), and then it was filtered again by a 0.22-μm filter (Zhao and Liang, 2019). Acetoxymethylester (BCECF-AM) was purchased from the Beyotime Institute of Biotechnology (Jiangsu, China).

### Monocyte–Endothelial Cell Adhesion Assay

HUVECs were seeded into 6-well plates and grown until almost 80% confluence. Then, the cells were incubated with dauricine at different concentrations for 8 h before 4-h stimulation with IL-1β (10 ng/ml). Before use, THP-1 cells were harvested and resuspended in 1640 medium after being labeled with BCECF-AM for approximately 30 min. The collected THP-1 cells were then added to each well containing HUVECs. After being incubated for 30 min, the suspended THP-1 cells were removed with phosphate-buffered saline (PBS). The fluorescence signals of each well were photographed and then analyzed.

### Real-Time Polymerase Chain Reaction Assay

Primary HUVECs were pretreated with dauricine (5, 10, 20, and 40 μM) for 8 h. IL-1β was then administered for 4 h to induce endothelial inflammation. After the treatment, HUVECs were harvested for RNA extraction using a TRIzol reagent (TaKaRa, Dalian, China; 9108). Total RAN was then transcribed into cDNA by a PCR kit (TaKaRa) according to the manufacturer’s protocol. Real-time PCR (RT-PCR) was conducted using commercial kits (TaKaRa, RR820Q) as instructed by the manufacturer. Relative mRNA expression levels were normalized and evaluated by the 2^−ΔΔCT^ method. The primers used for target genes are all listed in Supplementary Table S1.

### Western Blotting

Western blotting assays were carried out as described previously (Du et al., 2017). Primary antibodies in the experiments were applied as follows: ICAM-1 (diluted at 1:1,000, ProteinTech, Chicago, IL, United States), VCAM-1 (diluted at 1:1,000, ProteinTech), E-selectin (diluted at 1:1,000, R&D Systems, Minneapolis, MN, United States), β-tubulin (diluted at 1:1,000, ProteinTech), NF-κB-p65 (diluted at 1:1,000, CST, Danvers, MA, United States), NF-κB-pp65 (diluted at 1:1,000, CST), IκBα (diluted at 1:1,000, CST), and phospho-IκBα (diluted at 1:1,000, CST). Then the secondary antibodies with horseradish peroxidase coupling (1:10,000 dilution) obtained from ProteinTech were used for 2 h at room temperature. Electrochemiluminescence (ECL) detection reagents were purchased from Millipore (Billerica, MA, United States). Target proteins in the membrane were visualized with a Bio-Rad (Hercules, CA, United States) exposure imaging system.

### Nuclear and Cytoplasmic Protein Extraction

HUVECs were grown to 90% confluence in a 60-mm cell culture dish. Then vehicle (DMSO) or dauricine (40 μM) was given to the cells for 8 h. After that, IL-1β (10 ng/ml) was administered for another 4 h. Subsequently, cells were harvested for nuclear and cytosolic proteins extraction by using an extraction kit (Beyotime, P0027) according to the manufacturer’s instructions.

### Immunofluorescence

HUVECs were given a stimulation by IL-1β (10 ng/ml) for 30 min after the pretreatment with dauricine (5, 10, 20, and 40 μM) for 8 h. Cells were then harvested for immunofluorescence assay as previously described (Zhong et al., 2020). A specific antibody targeting p65 and a secondary antibody (Alexa Fluor®488, 1:800) were obtained from CST. Images were taken with a fluorescence microscope (Olympus, Tokyo, Japan). Immunofluorescence staining of paraffin-embedded lung sections was performed using a similar procedure after dewaxing and antigen recovery.

### Luciferase Reporter Assay

A p65-overexpressing vector and luciferase reporter gene vector containing the promoters for ICAM-1, VCAM-1, and E-selectin were constructed as described before (Zhao and Liang, 2019). Then, the vectors were introduced into 293T cells when the cell density reached 70%–80% confluence. After incubation for 24 h, the cells were administered with dauricine at a concentration of 40 μM for 8 h, and then cells were harvested. The Dual-Glo luciferase Assay System (Promega, Madison, WI, United States) was used to detect the activity of luciferase.

### Chromatin Immunoprecipitation Assay

Chromatin immunoprecipitation (ChIP) assay was carried out by a ChIP assay kit (Millipore). Rabbit IgG was used as the negative control. The fractured DNA samples were pulled down by an anti-NF-κB p65 antibody (CST). Subsequently, the enriched DNA fragments were detected by PCR using specific primers as shown in Supplementary Table S2 (Rao et al., 2019).

### Animal Experiment

All animal experiments were approved by the Animal Ethics Committee of Tongji Medical College, Huazhong University of Science and Technology. Male C57BL/6 mice (8–10 weeks) were obtained from Wuhan Beiente Biotechnology Company (Hubei, China). DMSO or dauricine was administered orally with a straight needle (12-gauge, 55 mm) once per day at the same time in seven successive days. LPS (15 mg/kg, Sigma) was then intraperitoneally injected to induce acute lung inflammation. The mice were euthanized to collect lung tissues 24 h after the administration of LPS.

### Statistical Analysis

All experiments were performed at least in triplicate. GraphPad Prism software was used for data analysis and output. ImageJ was used for the quantification of images from Western blotting assays. Quantitative data were presented as the mean ± standard error of mean (SEM). Student’s t-tests were performed to assess the statistical significance between two groups. *p* < 0.05 was considered to be statistically significant.

## Results

### Dauricine Was Nontoxic to Human Umbilical Vein Endothelial Cells

**FIGURE 1:**
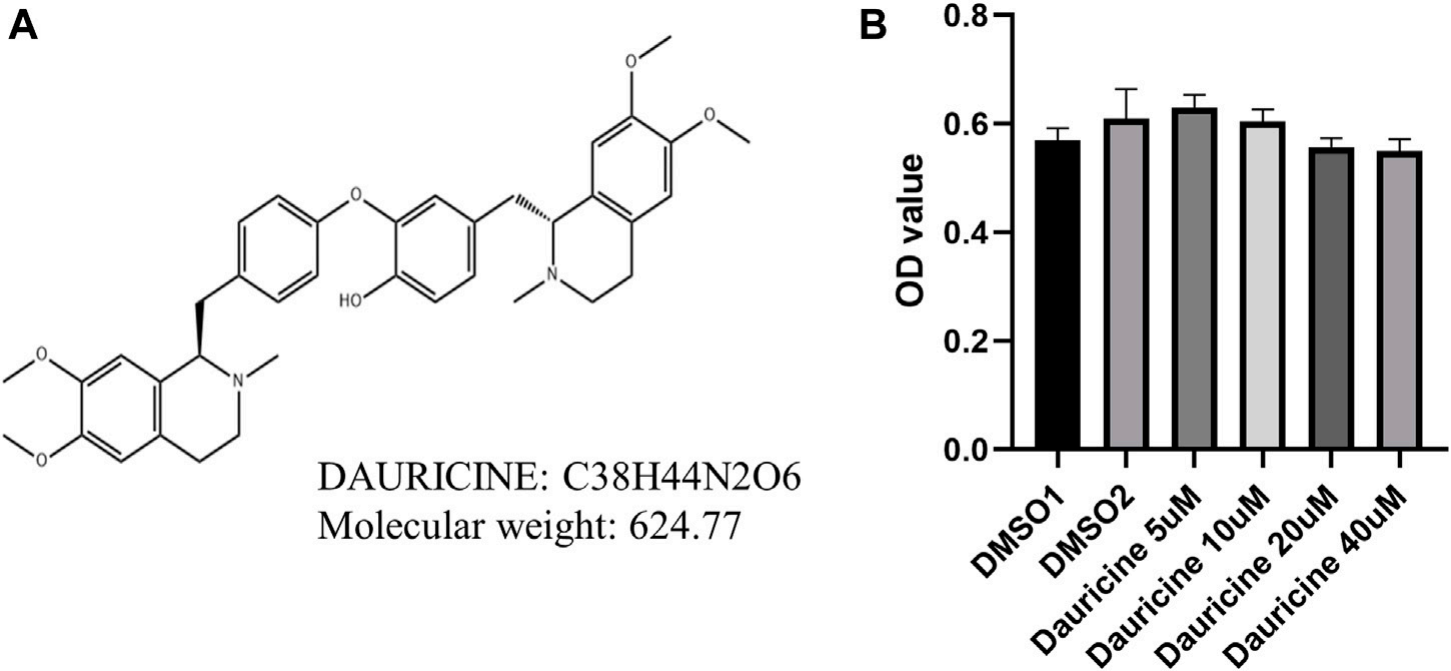
Chemical structure and cytotoxicity induced by dauricine. **(A)** Chemical structure of dauricine. **(B)** Dauricine was safe to use for HUVECs at concentrations of 0–40 μM, as determined by MTT assay (n = 5). HUVECs, human umbilical vein endothelial cells.

The chemical structural formula of dauricine is shown in Figure 1A. To investigate the effect of dauricine on endothelial cells, we first conducted the MTT assay to explore its cytotoxicity on HUVECs. As shown in Figure 1B, dauricine was safe to use for HUVECs at doses ranging from 5 to 40 μM.

### Dauricine Inhibited the Interactions Between Monocyte–Endothelial Cells

**FIGURE 2:**
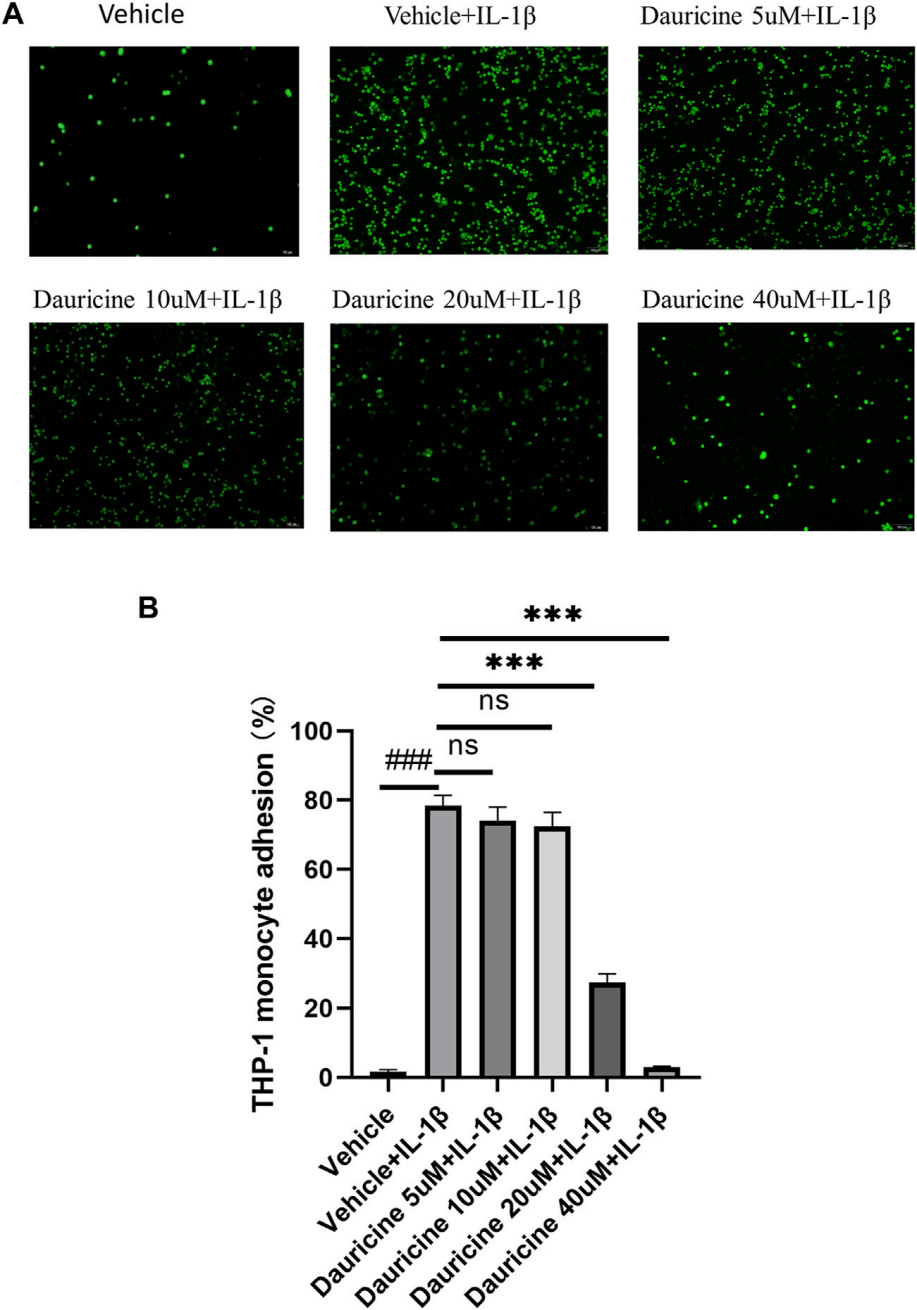
Dauricine inhibited monocyte–endothelial cell interaction. **(A)** Representative images of THP-1 cells (green) that adhered to HUVECs (scale bar: 100 μm). **(B)** Quantitative analysis of THP-1 cells that adhered to HUVECs. Data are expressed as mean ± SD. ###*p* < 0.001 versus the vehicle group; ***p < 0.001 versus the vehicle + IL-1β group. DMSO, dimethyl sulfoxide; IL-1β, interleukin-1β; HUVECs, human umbilical vein endothelial cells.

To explore the role of dauricine on IL-1β-induced endothelial inflammation, first, we assessed its function on monocyte–endothelial cell adhesion. An increased amount of THP-1 cells was attracted to HUVECs stimulated by IL-1β (10 ng/ml) in comparison with the nontreated HUVECs. However, when HUVECs were pretreated with dauricine, they attracted markedly fewer THP-1 cells (Figures 2A,B).

### Dauricine Decreased the Expression of Cell Adhesion Molecules

**FIGURE 3:**
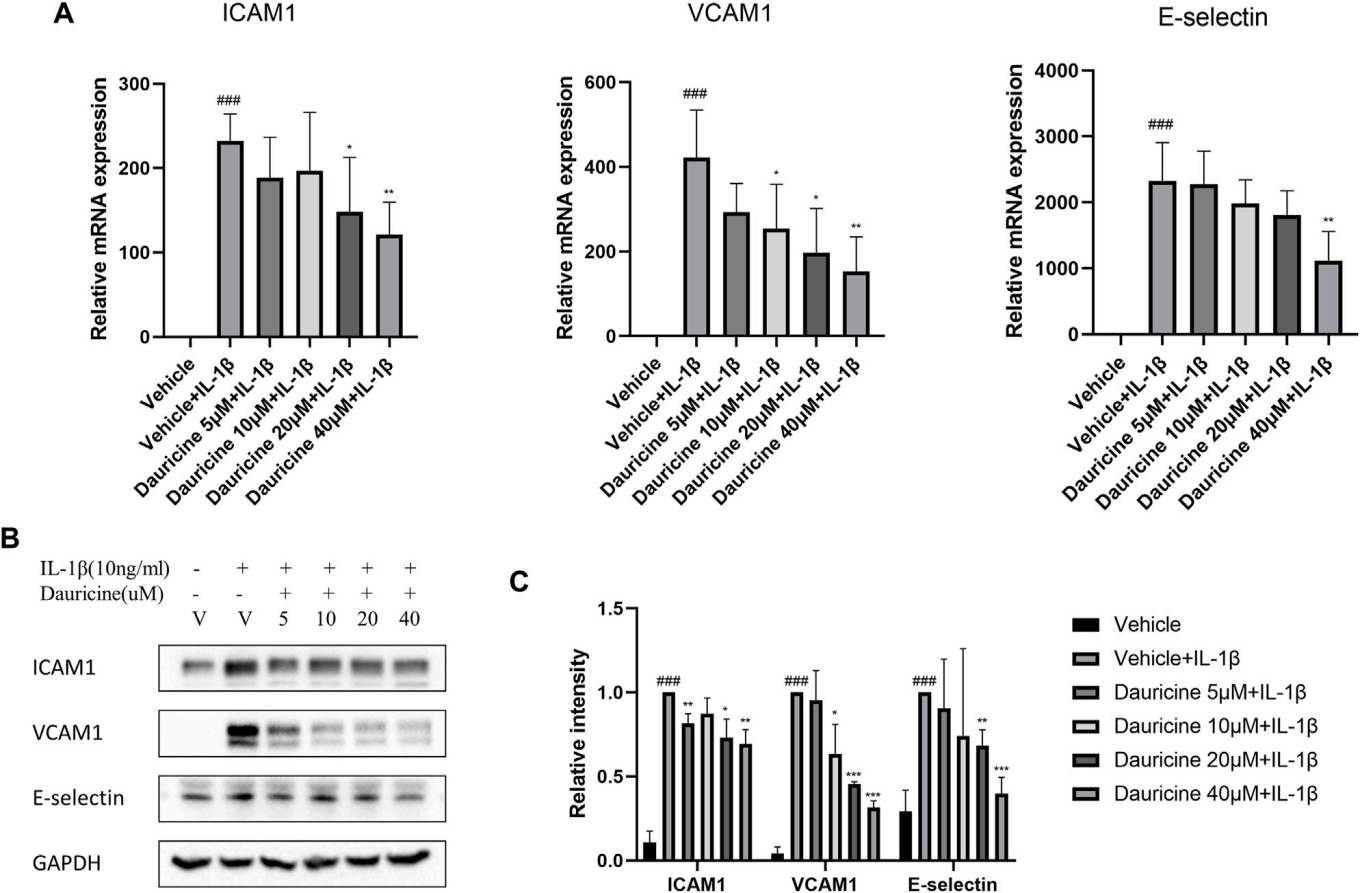
Dauricine decreased the expression of ICAM-1, VCAM-1, and E-selectin in HUVECs. **(A)** Relative mRNA expression levels of ICAM-1, VCAM-1, and E-selectin in HUVECs treated with IL-1β and dauricine by quantitative PCR (qPCR). **(B)** The protein levels of ICAM-1, VCAM-1, and E-selectin in HUVECs by Western blotting. **(C)** Quantification of the relative expression level of proteins in panel B. Data are expressed as mean ± SD. ###*p* < 0.001 versus the vehicle group; ***p < 0.001, **p < 0.01, *p < 0.05 versus the vehicle + IL-1β group. HUVECs, human umbilical vein endothelial cells; IL-1β, interleukin-1β.

Adhesion molecules are vital in the recruitment of inflammatory cells to endothelium. To further elucidate the role of dauricine on monocyte–endothelial cell interactions, we detected the expression levels of typical cell adhesion molecules in endothelial cells. The mRNA levels of ICAM-1, VCAM-1, and E-selectin were significantly upregulated in IL-1β-treated HUVECs (Figure 3A). However, treatment with dauricine suppressed the overexpression of these molecules (Figure 3A). Western blotting assays confirmed the decreased expression of ICAM-1, VCAM-1, and E-selectin in cells pretreated with dauricine (Figures 3B,C).

### Dauricine Inhibited IL-1β Induced Activation of NF-κB

**FIGURE 4:**
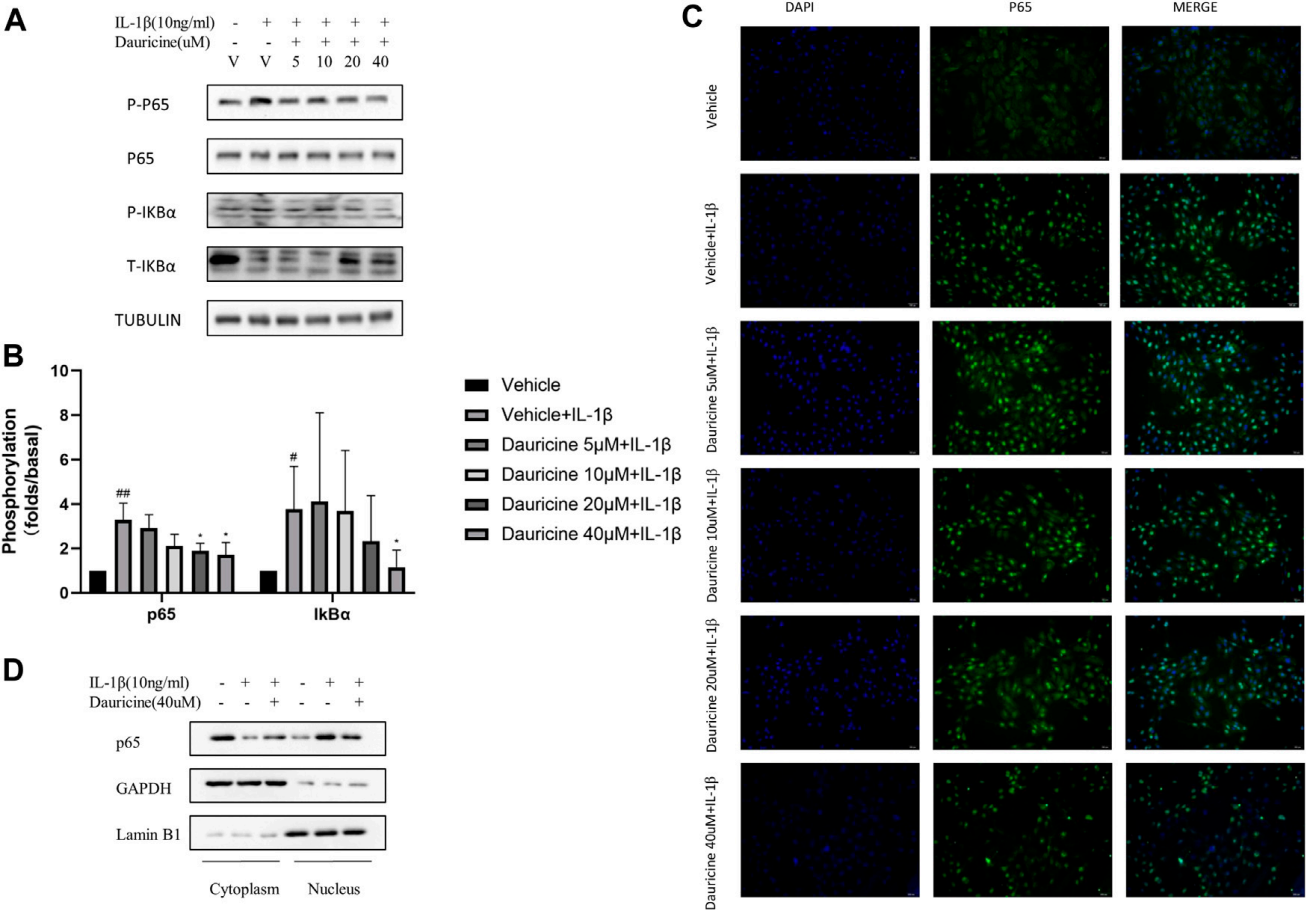
Dauricine inhibited IL-1β-induced NF-κB activation. **(A)** Phosphorylation of p65 and IκBα in HUVECs treated with IL-1β and dauricine at different concentrations. **(B)** Quantification of the phosphorylation of p65 and IκBα displayed in panel A. ##*p* < 0.01, #*p* < 0.05 versus the vehicle group; *p < 0.05 versus the vehicle + IL-1β group. **(C)** Nuclear translocation of p65, as indicated by the immunofluorescence in HUVECs treated with IL-1β and dauricine (scale bar: 200 μm). DAPI was used for nuclear staining (blue). P65 (green) was stained with the Alexa Fluor 488 secondary antibody. (D) Cytoplasmic and nuclear distribution of p65 in HUVECs as detected by Western blotting analysis. IL-1β, interleukin-1β; HUVECs, human umbilical vein endothelial cells.

The NF-κB pathway has been proven to regulate the expression of cell adhesion molecules in endothelial cells (Rao et al., 2019). To determine the mechanism how dauricine regulated the expression of ICAM-1, VCAM-1, and E-selectin, we detected the activation of NF-κB pathway in HUVECs stimulated with IL-1β. The degradation of IκBα and the activation of p65 were significantly inhibited by dauricine (Figures 4A,B). To further clarify the role of dauricine on p65 translocation, we detected the distribution of p65 protein by immunostaining. Correspondingly, we found that dauricine effectively inhibited p65 translocation in IL-1β-treated HUVECs (Figure 4C). Next, nuclear and cytoplasmic proteins were separately extracted to detect the level of p65 in both fractions. As shown in Figure 4D, the level of cytoplasmic p65 was decreased, and endonuclear p65 was significantly increased in IL-1β-treated HUVECs, while incubation with 40 μM of dauricine significantly inhibited the IL-1β-induced nuclear localization of p65.

### Dauricine Suppressed the Expression of ICAM-1, VCAM-1, and E-Selectin Through the NF-κB Pathway

**FIGURE 5:**
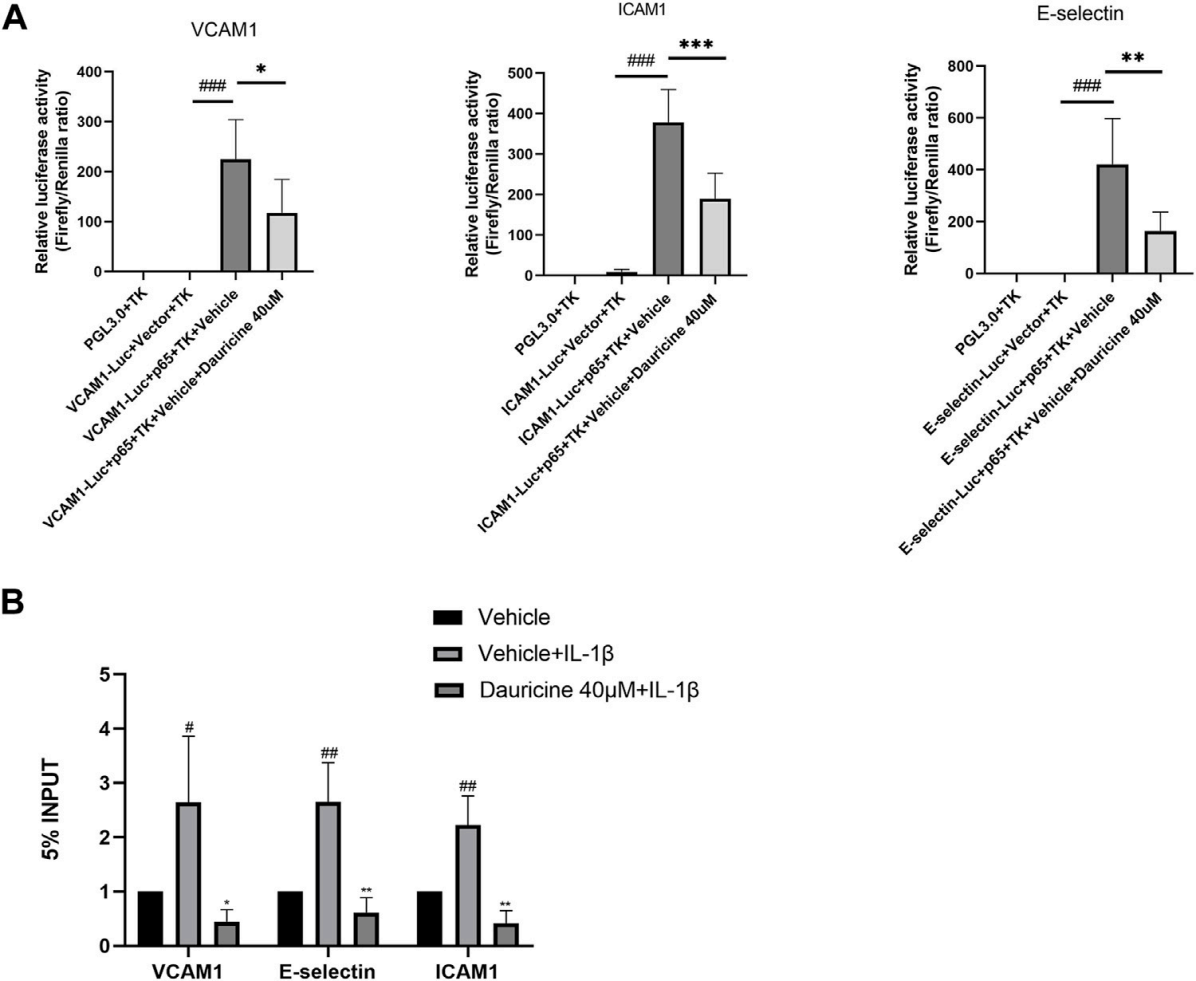
Dauricine suppressed NF-κB activation-induced expression of ICAM-1, VCAM-1, and E-selectin. **(A)** Dauricine inhibited ICAM-1, VCAM-1, and E-selectin promoter-derived luciferase activity individually in 293T cells cotransfected with a p65-overexpressing vector. ###*p* < 0.001 versus the VCAM-1, ICAM-1, E-selectin-Luc + empty vector + TK groups; ***p < 0.001, **p < 0.05 and *p < 0.05 versus the VCAM-1, ICAM-1, E-selectin-Luc + p65-expressing vector + vehicle + TK groups. **(B)** Dauricine reduced NF-κB p65 binding to its downstream gene promoters, including the ICAM-1, VCAM-1, and E-selectin promoters, as detected by ChIP assay. ChIP, chromatin immunoprecipitation.

Reporter gene constructs targeting ICAM-1, VCAM-1, and E-selectin were constructed for use in a dual-luciferase reporter assay. As shown in Figure 5A, the luciferase activities of E-selectin, ICAM-1, and VCAM-1 were obviously enhanced in 293T cells cotransfected with a p65 overexpression vector, while the administration of dauricine remarkably reduced their transcriptional activity. Furthermore, ChIP experiments were performed to determine the effect of dauricine on the DNA-binding activity of p65. Dauricine decreased the binding of p65 to its target gene promoters, including the promoters of VCAM-1, ICAM-1, and E-selectin, in IL-1β-treated endothelial cells (Figure 5B).

### Dauricine Attenuated Lipopolysaccharide-Induced Acute Lung Injury

**FIGURE 6:**
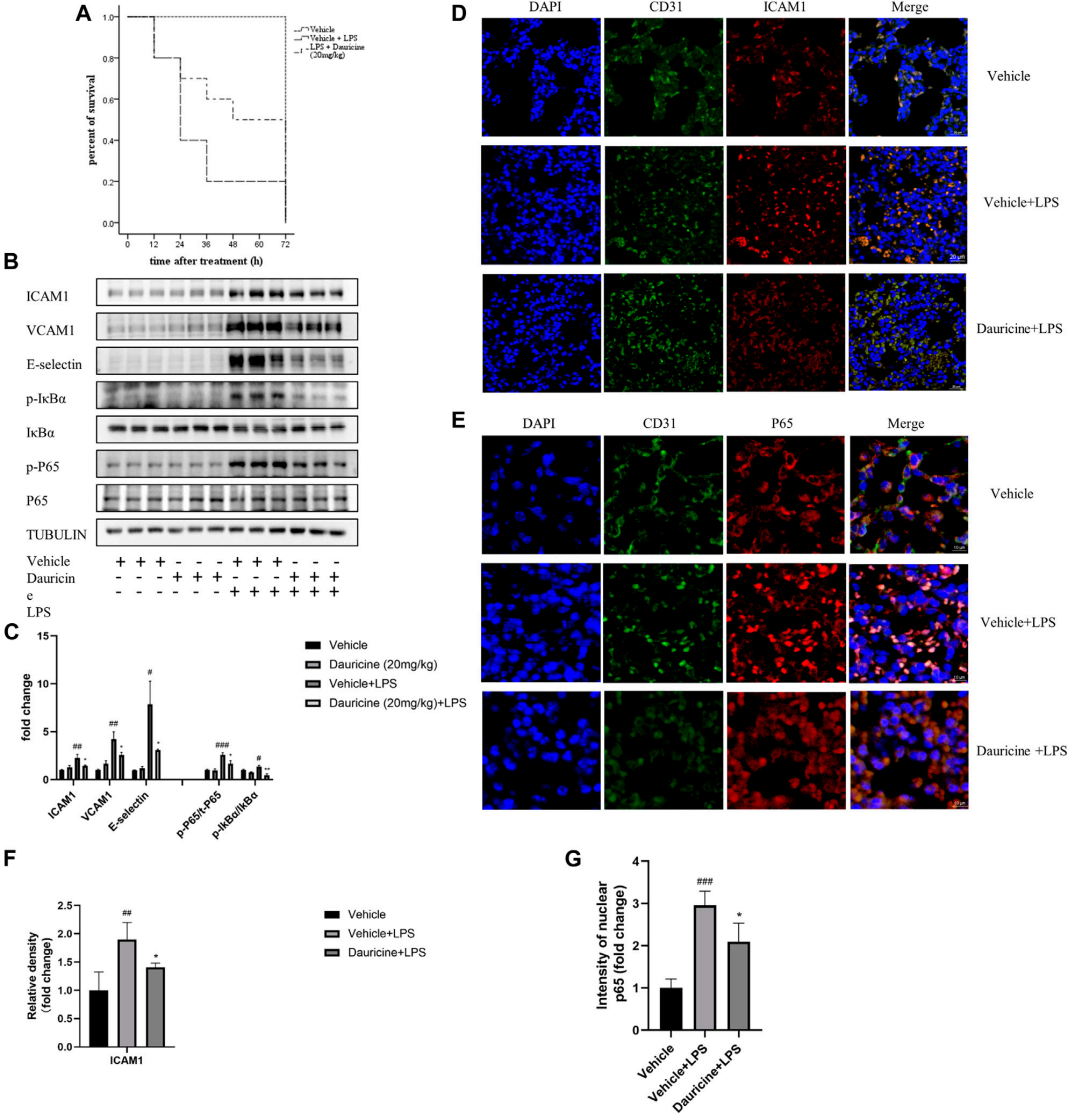
Dauricine attenuated LPS-induced acute lung injury *in vivo*. **(A)** Survival curves for mice treated with LPS and dauricine (20 mg/kg), n = 10/group. **(B)** Protein levels of ICAM-1, VCAM-1, and E-selectin and the phosphorylation of IκBα and p65 in lung tissues. **(C)** Quantification analysis of the protein levels in panel B. ###*p* < 0.001, ##*p* < 0.01, #*p* < 0.05 versus the vehicle group; **p < 0.01, *p < 0.05 versus the vehicle + LPS group. **(D)** Level of endothelial cell-expressed ICAM-1 in lung tissues, as detected by immunofluorescence assay (scale bar: 20 μm). DAPI was used for nucleus staining (blue), ICAM-1 was stained red, and CD31 was stained green. **(E)** Nuclear translocation of p65 in endothelial cells as detected by immunofluorescence assay in lung tissues (scale bar: 10 μm). DAPI was used for nucleus staining (blue), p65 was stained red, and CD31 was stained green. **(F)** Quantification analysis of ICAM-1 in panel D. ##*p* < 0.01 versus the vehicle group; *p < 0.05 versus the vehicle + LPS group. **(G)** Quantification analysis of nucleic p65 in panel E. ###*p* < 0.001 versus the vehicle group; *p < 0.05 versus the vehicle + LPS group. LPS, lipopolysaccharide.

**FIGURE 7:**
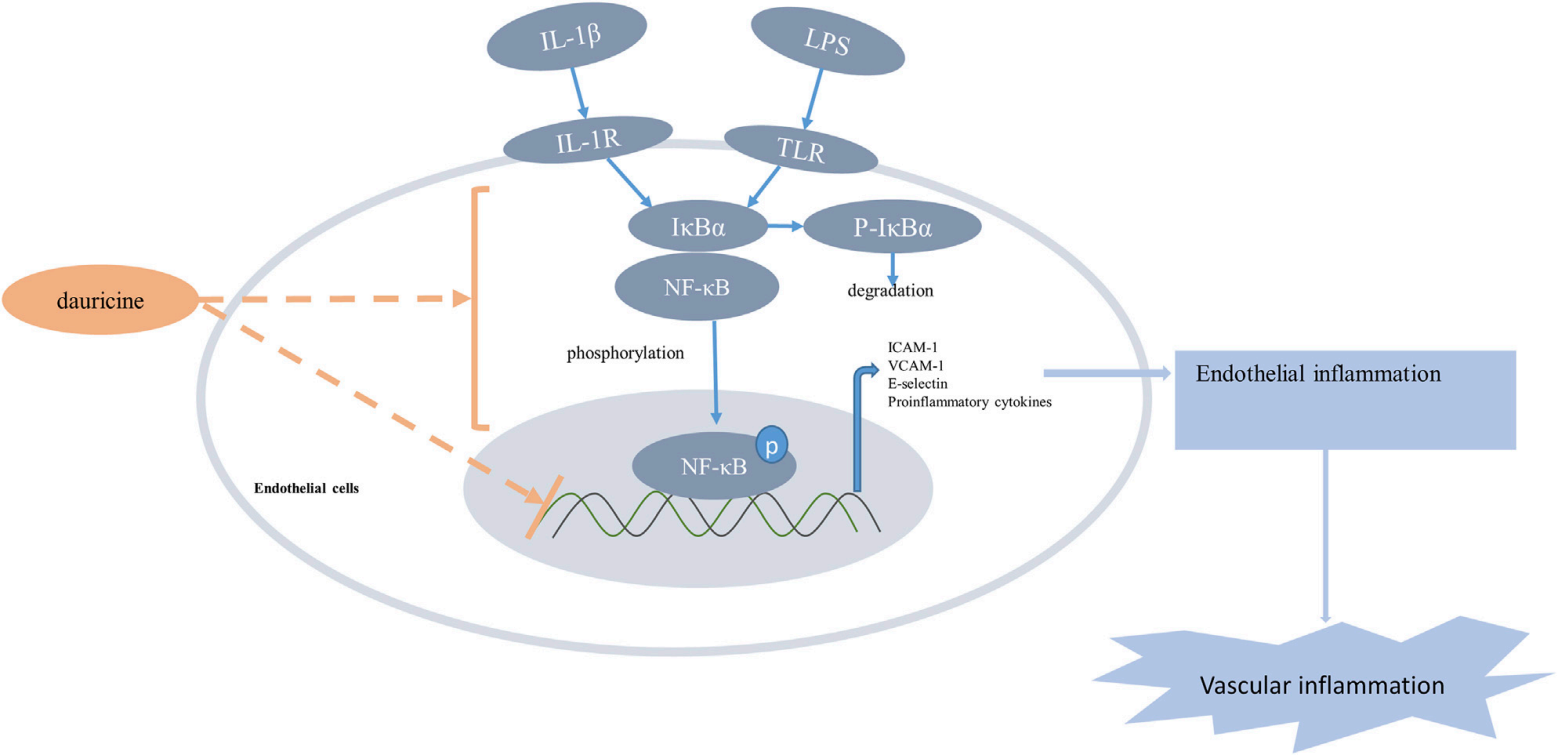
Schematic illustration of the anti-inflammatory effect of dauricine. Proinflammatory stimulus such as LPS and IL-1β increased the phosphorylation and nuclear translocation of the NF-κB p65 subunit, which resulted in the upregulation of adhesion molecules and many other inflammatory cytokines that could aggravate vascular inflammation. Dauricine showed an inhibition of NF-kB pathway and downregulated the expression of its downstream genes to attenuate endothelial inflammation. IL-1β, interleukin-1β; LPS, lipopolysaccharide.

Based on previous studies that demonstrated the anti-inflammatory role of dauricine in IL-1β-treated endothelial cells, we established an acute lung injury model induced by LPS to further verify whether dauricine attenuated endothelial inflammation *in vivo*. Previously, we reported that dauricine alleviated LPS-induced macrophage infiltration in lung tissues and decreased serum levels of IL-1β, IL-6, and TNF-α (Qiao et al., 2019). In this work, we found that pretreatment with dauricine (20 mg/kg) effectively protected mice from LPS-induced acute inflammation (Figure 6A). Moreover, the expression of ICAM-1, VCAM-1, and E-selectin in lung tissues was decreased by dauricine pretreatment in LPS-treated mice (Figures 6B,C). Additionally, the phosphorylation of IκBα and p65 was also inhibited (Figures 6B,C). We performed immunofluorescence assay on lung tissues to determine the levels of ICAM-1, VCAM-1, and E-selectin expressed in endothelial cells. The expression of ICAM-1 was increased in the endothelial cells of lung tissues from LPS-treated mice, while it was attenuated in dauricine-treated mice (Figures 6D,F). Similar results were observed in the level of VCAM-1 and E-selectin (Supplementary figure S1A,B,C). Moreover, we found that p65 translocation in lung endothelial cells was significantly reduced by dauricine pretreatment compared with the LPS-treated group (Figures 6E,G). We concluded that dauricine could alleviate the inflammatory response in lung endothelial cells, which corresponded with our *in vitro* findings (Figure 7).

## Discussion

The relationship between endothelial inflammation and various diseases, including metabolic syndrome, chronic kidney disease, rheumatoid arthritis, and sepsis, has been debated for a long time (Yang et al., 2016; Grandl and Wolfrum, 2018; Diaz-Ricart et al., 2020; Lupu et al., 2020). During vascular inflammation, the expression of adhesion molecules, including ICAM-1, VCAM-1, and E-selectin, is elevated, promoting leukocyte recruitment to the endothelium (Zhao and Liang, 2019). Excessive leukocyte recruitment to the endothelium aggravates local inflammation and contributes to endothelial inflammation-related diseases such as atherosclerosis and dysfunctional microcirculation (Hernandez et al., 2013; Schmitt et al., 2014). Molecules and drugs targeting endothelial inflammation may be potential candidates for various similar diseases.

Traditional Chinese medicine has been used in inflammatory diseases for a long time (Ren et al., 2017; Chiang et al., 2020). And in recent years, many extracts from traditional herbs were proved to regulate several signaling pathways involved in inflammation (Spelman et al., 2006; Huang et al., 2016; Zhang et al., 2019). Dauricine, an alkaloid isolated from *M. dauricum*, has been found to protect against several inflammation-related diseases. Previous studies have reported that dauricine inhibited NF-κB activation in macrophages and colon cancer cells (Yang et al., 2010; Qiao et al., 2019). In a cerebral ischemia/reperfusion rat model treated with dauricine, ICAM-1 was proven to be significantly reduced. In this work, we demonstrated that dauricine repressed IL-1β-induced NF-κB activation and reduced the expression of its downstream genes in endothelial cells. Therefore, dauricine could be considered for use in diseases related with endothelial inflammation.

The NF-κB pathway is vital to endothelial inflammation (Oates, 2015). Usually, the activation of cytoplasmic NF-κB is regulated by inhibitors of κB (IκB). Extracellular proinflammatory stimulus promotes the phosphorylation and degradation of IκB; subsequently, the active unit, P65, is translocated to the nucleus, where it regulates the expression of downstream genes (Lee et al., 2009). However, the mechanism by which dauricine regulates the activation of the NF-κB pathway remains unknown. Further study is needed to determine whether dauricine regulates NF-κB directly by binding with its subunit or through other pathways.

Endothelial cells play pivotal roles in LPS-induced inflammation (Flavahan, 1992). We tested the anti-inflammatory function of dauricine in a mouse model of acute lung injury induced by LPS. We showed that mice pretreated with dauricine exhibited dramatically decreased expression of ICAM-1, VCAM-1, and E-selectin in lung vascular beds. Activation of the NF-κB pathway was also downregulated in lung tissue after pretreatment with dauricine. These findings suggested that dauricine reduced acute lung inflammation by inhibiting the NF-κB pathway. However, whether dauricine can prevent endothelial inflammation in other vascular-rich organs, such as the kidney, or in other inflammatory diseases, such as atherosclerosis, remains to be explored.

In addition, we found that MAPK pathways (the p38, ERK1/2, and JNK pathways) were activated in IL-1β-treated HUVECs and that dauricine could inhibit the phosphorylation of P38, JNK, and ERK (data not shown). MAPK pathways are ubiquitous and have been reported to regulate the expression of adhesion molecules, including ICAM-1, VCAM-1, and E-selectin in inflammatory diseases (Min and Pober, 1997; Pietersma et al., 1997; Yan et al., 2002). Therefore, dauricine might also exert its anti-inflammatory effect by inhibiting the MAPK pathway. However, whether dauricine could alleviate the activation of the MAPK pathway *in vivo* and to what extent the MAPK and NF-κB pathway take part individually in the anti-inflammatory effect of dauricine remain unknown.

Collectively, the findings from our present study suggest that dauricine suppresses endothelial inflammation through or partially through inhibition of the NF-κB pathway. We propose that dauricine might serve as a potential therapeutic candidate for various diseases related to endothelial inflammation.

## Data Availability

The original contributions presented in the study are included in the article/Supplementary Material, Further inquiries can be directed to the corresponding author.

## References

[B1] Boulanger C. M. (2016). Endothelium. Arterioscler Thromb. Vasc. Biol. 36 (4), e26–31. 10.1161/atvbaha.116.306940 27010027

[B2] Brasier A. R. (2010). The Nuclear Factor-kappaB-Interleukin-6 Signalling Pathway Mediating Vascular Inflammation. Cardiovasc. Res. 86 (2), 211–218. 10.1093/cvr/cvq076 20202975 PMC2912657

[B3] Chiang C. C. Cheng W. J. Lin C. Y. Lai K. H. Ju S. C. Lee C. (2020). Kan-Lu-Hsiao-Tu-Tan, a Traditional Chinese Medicine Formula, Inhibits Human Neutrophil Activation and Ameliorates Imiquimod-Induced Psoriasis-like Skin Inflammation. J. Ethnopharmacol 246, 112246. 10.1016/j.jep.2019.112246 31539577

[B4] Diaz-Ricart M. Torramade-Moix S. Pascual G. Palomo M. Moreno-Castaño A. B. Martinez-Sanchez J. (2020). Endothelial Damage, Inflammation and Immunity in Chronic Kidney Disease. Toxins (Basel) 12 (6). 10.3390/toxins12060361 PMC7354562 32492843

[B5] Du M. Yuan L. Tan X. Huang D. Wang X. Zheng Z. (2017). The LPS-Inducible lncRNA Mirt2 Is a Negative Regulator of Inflammation. Nat. Commun. 8 (1), 2049. 10.1038/s41467-017-02229-1 29230038 PMC5725456

[B6] Du Z. H. Liu H. G. Chai C. Y. Luo L. Y. Hu C. J. (1986). Anti-inflammatory Effect of Dauricine. Zhongguo Yao Li Xue Bao 7 (5), 419–422. 2954413

[B7] Flavahan N. A. (1992). Atherosclerosis or Lipoprotein-Induced Endothelial Dysfunction. Potential Mechanisms Underlying Reduction in EDRF/nitric Oxide Activity. Circulation 85 (5), 1927–1938. 10.1161/01.cir.85.5.1927 1572048

[B8] Gimbrone M. A. Jr. García-Cardeña G. (2016). Endothelial Cell Dysfunction and the Pathobiology of Atherosclerosis. Circ. Res. 118 (4), 620–636. 10.1161/circresaha.115.306301 26892962 PMC4762052

[B9] Grandl G. Wolfrum C. (2018). Hemostasis, Endothelial Stress, Inflammation, and the Metabolic Syndrome. Semin. Immunopathol 40 (2), 215–224. 10.1007/s00281-017-0666-5 29209827 PMC5809518

[B10] Hernandez G. Bruhn A. Ince C. (2013). Microcirculation in Sepsis: New Perspectives. Curr. Vasc. Pharmacol. 11 (2), 161–169. 10.2174/1570161111311020006 23506495

[B11] Huang Y. Cai T. Xia X. Cai Y. Wu X. Y. (2016). Research Advances in the Intervention of Inflammation and Cancer by Active Ingredients of Traditional Chinese Medicine. J. Pharm. Pharm. Sci. 19 (1), 114–126. 10.18433/j3sg7k 27096696

[B12] Joffre J. Hellman J. Ince C. Ait-Oufella H. (2020). Endothelial Responses in Sepsis. Am. J. Respir. Crit. Care Med. 202 (3), 361–370. 10.1164/rccm.201910-1911TR 32101446

[B13] Jourde-Chiche N. Fakhouri F. Dou L. Bellien J. Burtey S. Frimat M. (2019). Endothelium Structure and Function in Kidney Health and Disease. Nat. Rev. Nephrol. 15 (2), 87–108. 10.1038/s41581-018-0098-z 30607032

[B14] Konukoglu D. Uzun H. (2017). Endothelial Dysfunction and Hypertension. Adv. Exp. Med. Biol. 956, 511–540. 10.1007/5584_2016_90 28035582

[B15] Lee Y. M. Seon M. R. Cho H. J. Kim J. S. Park J. H. (2009). Benzyl Isothiocyanate Exhibits Anti-inflammatory Effects in Murine Macrophages and in Mouse Skin. J. Mol. Med. (Berl) 87 (12), 1251–1261. 10.1007/s00109-009-0532-6 19760383

[B16] Li H. Chen X. Zhou S. J. (2018). Dauricine Combined with Clindamycin Inhibits Severe Pneumonia Co-infected by Influenza Virus H5N1 and Streptococcus Pneumoniae In vitro and In vivo through NF-Κb Signaling Pathway. J. Pharmacol. Sci. 137 (1), 12–19. 10.1016/j.jphs.2018.01.011 29769163

[B17] Lupu F. Kinasewitz G. Dormer K. (2020). The Role of Endothelial Shear Stress on Haemodynamics, Inflammation, Coagulation and Glycocalyx during Sepsis. J. Cel Mol Med 24 (21), 12258–12271. 10.1111/jcmm.15895 PMC7687012 32951280

[B18] Min W. Pober J. S. (1997). TNF Initiates E-Selectin Transcription in Human Endothelial Cells through Parallel TRAF-NF-Kappa B and TRAF-RAC/CDC42-JNK-c-Jun/ATF2 Pathways. J. Immunol. 159 (7), 3508–3518. 9317150

[B19] Oates J. C. (2015). Endothelial Dysfunction in Injury and Inflammation. Am. J. Med. Sci. 349 (1), 2. 10.1097/maj.0000000000000401 25535885 PMC6314289

[B20] Park H. J. Gholam Zadeh M. Suh J. H. Choi H. S. (2020). Dauricine Protects from LPS-Induced Bone Loss via the ROS/PP2A/NF-κB Axis in Osteoclasts. Antioxidants (Basel) 9 (7), 588. 10.3390/antiox9070588 32640590 PMC7402093

[B21] Pietersma A. Tilly B. C. Gaestel M. de Jong N. Lee J. C. Koster J. F. (1997). p38 Mitogen Activated Protein Kinase Regulates Endothelial VCAM-1 Expression at the post-transcriptional Level. Biochem. Biophys. Res. Commun. 230 (1), 44–48. 10.1006/bbrc.1996.5886 9020057

[B22] Qiao B. Wang H. Wang C. Liang M. Huang K. Li Y. (2019). Dauricine Negatively Regulates Lipopolysaccharide- or Cecal Ligation and Puncture-Induced Inflammatory Response via NF-Κb Inactivation. Arch. Biochem. Biophys. 666, 99–106. 10.1016/j.abb.2019.03.018 30946805

[B23] Rao C. Liu B. Huang D. Chen R. Huang K. Li F. (2021). Nucleophosmin Contributes to Vascular Inflammation and Endothelial Dysfunction in Atherosclerosis Progression. J. Thorac. Cardiovasc. Surg. 161, e377–e393. 10.1016/j.jtcvs.2019.10.152 32007256

[B24] Ren Y. Qiao W. Fu D. Han Z. Liu W. Ye W. (2017). Traditional Chinese Medicine Protects against Cytokine Production as the Potential Immunosuppressive Agents in Atherosclerosis. J. Immunol. Res. 2017, 7424307. 10.1155/2017/7424307 29038791 PMC5606136

[B25] Schmitt M. M. Megens R. T. Zernecke A. Bidzhekov K. van den Akker N. M. Rademakers T. (2014). Endothelial Junctional Adhesion Molecule-A Guides Monocytes into Flow-dependent Predilection Sites of Atherosclerosis. Circulation 129 (1), 66–76. 10.1161/circulationaha.113.004149 24065611

[B26] Spelman K. Burns J. Nichols D. Winters N. Ottersberg S. Tenborg M. (2006). Modulation of Cytokine Expression by Traditional Medicines: a Review of Herbal Immunomodulators. Altern. Med. Rev. 11 (2), 128–150. 16813462

[B27] Sturtzel C. (2017). Endothelial Cells. Adv. Exp. Med. Biol. 1003, 71–91. 10.1007/978-3-319-57613-8_4 28667554

[B28] Yan W. Zhao K. Jiang Y. Huang Q. Wang J. Kan W. (2002). Role of P38 MAPK in ICAM-1 Expression of Vascular Endothelial Cells Induced by Lipopolysaccharide. Shock 17 (5), 433–438. 10.1097/00024382-200205000-00016 12022767

[B29] Yang X. Chang Y. Wei W. (2016). Endothelial Dysfunction and Inflammation: Immunity in Rheumatoid Arthritis. Mediators Inflamm. 2016, 6813016. 10.1155/2016/6813016 27122657 PMC4829719

[B30] Yang X. Y. Jiang S. Q. Zhang L. Liu Q. N. Gong P. L. (2007). Inhibitory Effect of Dauricine on Inflammatory Process Following Focal Cerebral Ischemia/reperfusion in Rats. Am. J. Chin. Med. 35 (3), 477–486. 10.1142/s0192415x07004990 17597506

[B31] Yang Z. Li C. Wang X. Zhai C. Yi Z. Wang L. (2010). Dauricine Induces Apoptosis, Inhibits Proliferation and Invasion through Inhibiting NF-kappaB Signaling Pathway in colon Cancer Cells. J. Cel Physiol 225 (1), 266–275. 10.1002/jcp.22261 20509140

[B32] Zhang J. Zhang Q. Liu G. Zhang N. (2019). Therapeutic Potentials and Mechanisms of the Chinese Traditional Medicine Danshensu. Eur. J. Pharmacol. 864, 172710. 10.1016/j.ejphar.2019.172710 31586468

[B33] Zhang S. Ren Y. Qiu J. (2018). Dauricine Inhibits Viability and Induces Cell Cycle Arrest and Apoptosis via Inhibiting the PI3K/Akt Signaling Pathway in Renal Cell Carcinoma Cells. Mol. Med. Rep. 17 (5), 7403–7408. 10.3892/mmr.2018.8732 29568902

[B34] Zhao S. Liang M. Wang Y. Hu J. Zhong Y. Li J. (2019). Chrysin Suppresses Vascular Endothelial Inflammation via Inhibiting the NF-Κb Signaling Pathway. J. Cardiovasc. Pharmacol. Ther. 24 (3), 278–287. 10.1177/1074248418810809 30497287

[B35] Zhong Y. He K. Huang M. Liang M. (2020). Neferine Suppresses Vascular Endothelial Inflammation by Inhibiting the NF-Κb Signaling Pathwayy. Arch. Biochem. Biophys. 696, 108595. 10.1016/j.abb.2020.108595 33157101

