# Corrigendum: Dauricine attenuates vascular endothelial inflammation through inhibiting NF-κB pathway

**DOI:** 10.3389/fphar.2023.1236892

**Published:** 2023-08-17

**Authors:** Ji Hu, Ru Chen, Jie An, Yilong Wang, Minglu Liang, Kai Huang

**Affiliations:** ^1^ Department of Cardiology, Union Hospital, Tongji Medical College, Huazhong University of Science and Technology, Wuhan, China; ^2^ Clinic Center of Human Gene Research, Union Hospital, Tongji Medical College, Huazhong University of Science and Technology, Wuhan, China; ^3^ Department of Cardiology, Handan First Hospital, Handan, China; ^4^ Department of Cardiology, The First Affiliated Hospital of Sun Yat-Sen University, Guangzhou, China; ^5^ Hubei Key Laboratory of Metabolic Abnormalities and Vascular Aging, Huazhong University of Science and Technology, Wuhan, China; ^6^ Hubei Clinical Research Center of Metabolic and Cardiovascular Disease, Huazhong University of Science and Technology, Wuhan, China

**Keywords:** dauricine, endothelial dysfunction, NF-κB pathway, acute lung injury, inflammation

In the published article, there was an error regarding the **Affiliation(s)** for “Kai Huang.” In addition to having **Affiliation(s)** 1 and 2, “[5] Hubei Key Laboratory of Metabolic Abnormalities and Vascular Aging, Huazhong University of Science and Technology, Wuhan, China; [6] Hubei Clinical Research Center of Metabolic and Cardiovascular Disease, Huazhong University of Science and Technology, Wuhan, China” should also be included.

In the published article, there was an error in Figure 4C, as published. Images of the dauricine-treated (40 um + IL-1β) group in Figure 4C were jumbled with the dauricine-treated (20 um + IL-1β) group. The corrected (Figure 4C) and its caption “(C) Nuclear translocation of p65, as indicated by the immunofluorescence in HUVECs treated with IL-1β and dauricine (scale bar: 200 μm). DAPI was used for nuclear staining (blue). P65 (green) was stained with the Alexa Fluor 488 secondary antibody” appear as follows:

**FIGURE 4:**
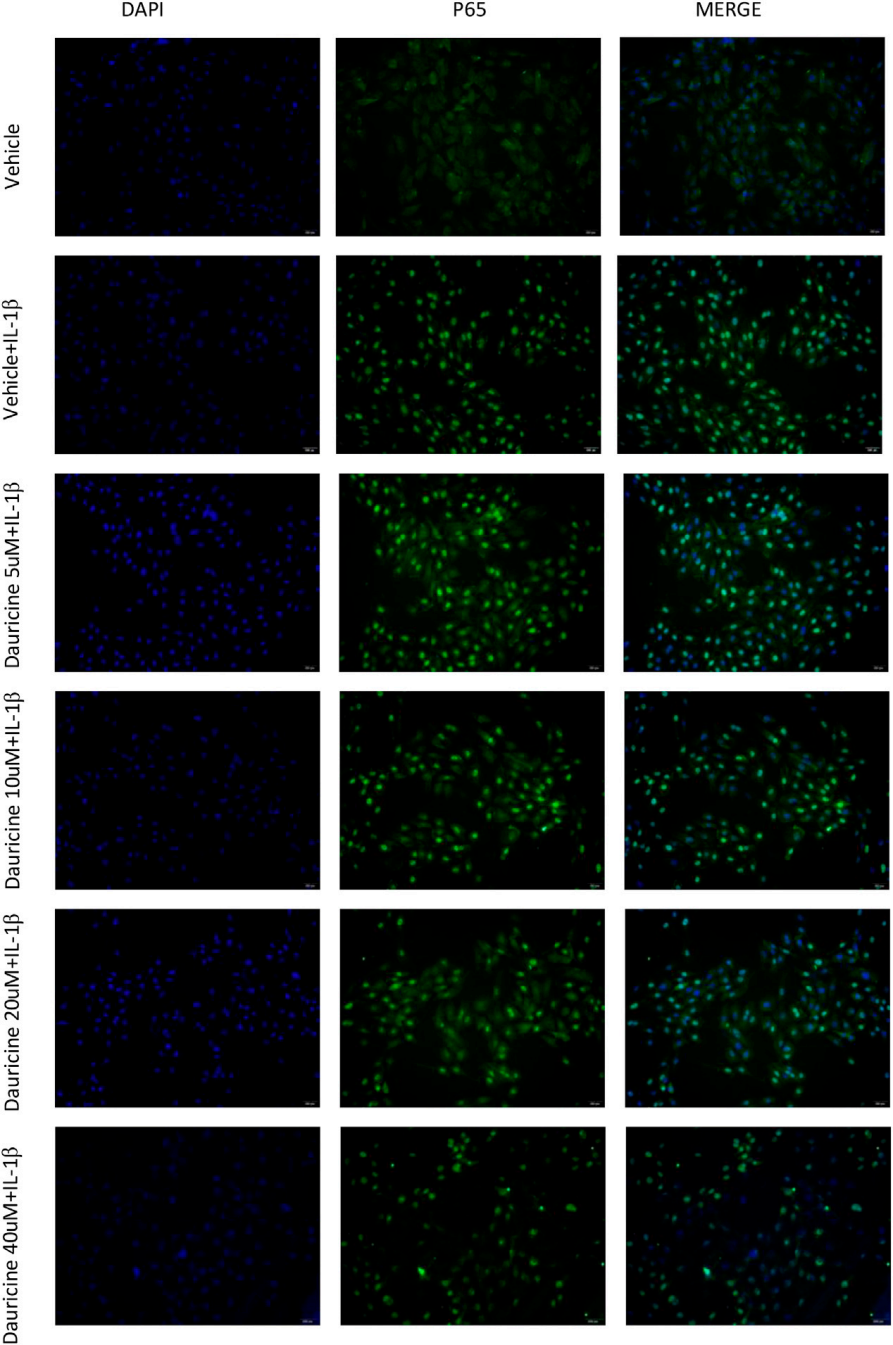
**(C)** Nuclear translocation of p65 as indicated by the immunofluorescence in HUVECs treated with IL-1β and dauricine (scale bar :200 μm). DAPI was used for nucleus staining (blue). P65 (green) was stained with a secondary antibody of Alexa Fluor 488.

Figure 4 (C) Nuclear translocation of p65 as indicated by the immunofluorescence in HUVECs treated with IL-1β and dauricine (scale bar :200 μm). DAPI was used for nucleus staining (blue). P65 (green) was stained with a secondary antibody of Alexa Fluor 488.

In the published article, there was an error in the **Funding** statement. The funding information was missing in the initial version. The correct **Funding** statement appears as follows:

“This study was supported by the National Natural Science Foundation of China (Nos: 81830014 and 91949201).”

The authors apologize for these errors and state that this does not change the scientific conclusions of the article in any way. The original article has been updated.

