# Supplementary material for: Dauricine Attenuates Vascular Endothelial Inflammation Through Inhibiting NF-κB Pathway
Source: Front Pharmacol. 2021 Dec 1;12:758962. doi: 10.3389/fphar.2021.758962 (PMC8672219; doi:10.3389/fphar.2021.758962)
Supplement: Supplementary file 1 [file Table1.docx]

| Supplementary table 1 real-time PCR primer sequences | |  |
| --- | --- | --- |
| Gene | Forward primer | Reverse primer |
| ICAM-1 | ATGGCAACGACTCCTTCTCG | GCCGGAAAGCTGTAGATGGT |
| VCAM-1 | TGGATAATGTTTGCAGCTTCTCA | CGTCACCTTCCCATTCAGTG |
| E-selectin | TGTGAGATGCGATGCTGTC | AACCTCTTCTGTCCATTGTCC |
| GAPDH | AATGGGCAGCCGTTAGGAAA | GCCCAATACGACCAAATCAGAG |
| 18s | TTGACGGAAGGGCACCACCAG | GCACCACCACCCACGGAATCG |
| ICAM-1, intercellular adhesion molecule-1; VCAM-1, vascular cell adhesion molecule-1; GAPDH, glyceraldehyde-3-phosphate dehydrogenase | | |
| Supplementary table 2 Primers used for chromatin immunoprecipitation assays | | |
| Gene | Forward primer | Reverse primer |
| ICAM-1 | CACTCCCACGGTTAGCGG | CCATTTCACAAAGCGGTAAAC |
| VCAM-1 | CAAGGTACCTTTATCTTTCCAGT  AAAGATAGCC | GATAGCTTAGCTCCTGAAGCC  AGTGAG |
| E-selectin | GGGAAAGTTTTTGGATGCCATT | TGTCCACATCCAGTAAAGAGGAAAT |
