# Supplementary figures and images for: Dauricine Attenuates Vascular Endothelial Inflammation Through Inhibiting NF-κB Pathway
Source: Front Pharmacol. 2021 Dec 1;12:758962. doi: 10.3389/fphar.2021.758962 (PMC8672219; doi:10.3389/fphar.2021.758962)

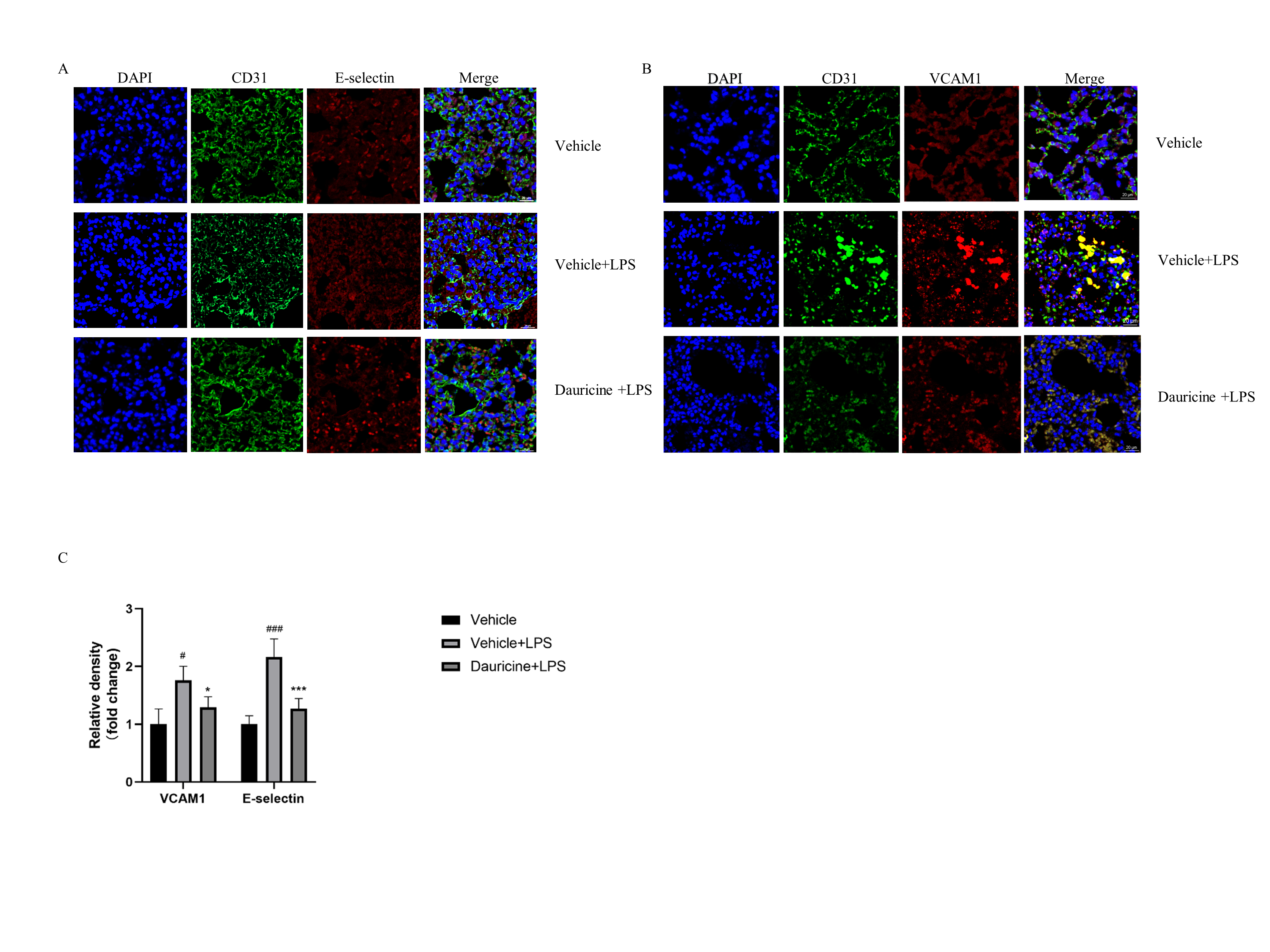

Supplement: Supplementary file 2 [file Image1.tif]
